# Growth of κ-([Al,In]_*x*_Ga_1-x_)_2_O_3_ Quantum Wells and Their Potential for Quantum-Well Infrared Photodetectors

**DOI:** 10.1021/acsami.3c02695

**Published:** 2023-06-06

**Authors:** Thorsten Schultz, Max Kneiß, Philipp Storm, Daniel Splith, Holger von Wenckstern, Christoph T. Koch, Adnan Hammud, Marius Grundmann, Norbert Koch

**Affiliations:** † Helmholtz-Zentrum Berlin für Materialien und Energie GmbH, Berlin 14109, Germany; ‡ Humboldt-Universität zu Berlin, Institut für Physik & IRIS Adlershof, Berlin 12489, Germany; § Universität Leipzig, Felix-Bloch-Institut für Festkörperphysik, Leipzig 04103, Germany; ∥ Department of Inorganic Chemistry, Fritz-Haber Institute of the Max-Planck Society, Berlin 14195, Germany

**Keywords:** pulsed laser deposition, X-ray photoelectron spectroscopy depth profiling, X-ray diffraction, transmission electron microscopy, heterostructures, interface analysis

## Abstract

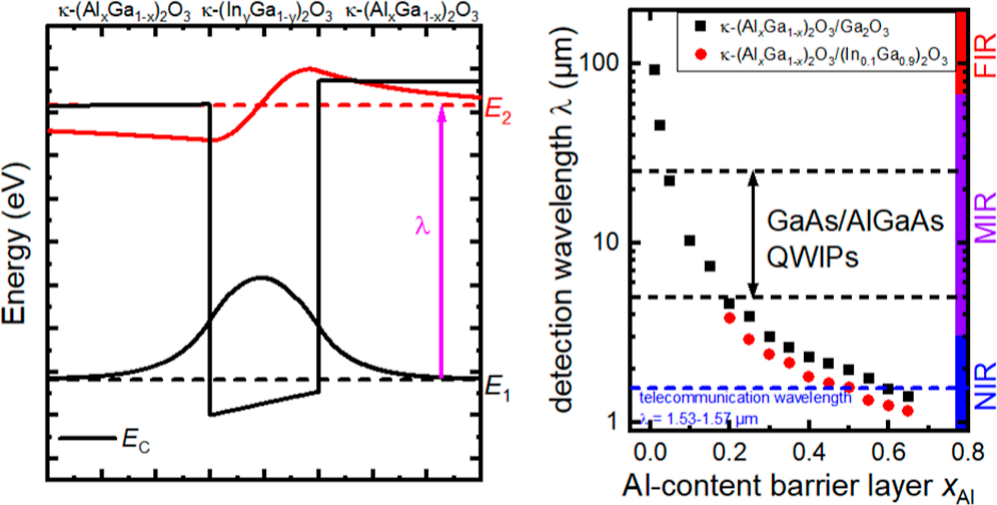

The wide band gap semiconductor κ-Ga_2_O_3_ and its aluminum and indium alloys have been proposed as promising materials for many applications. One of them is the use of inter-sub-band transitions in quantum-well (QW) systems for infrared detectors. Our simulations show that the detection wavelength range of nowadays state of the art GaAs/Al_*x*_Ga_1-*x*_As quantum-well infrared photodetectors (QWIPs) could be substantially excelled with about 1–100 μm using κ-([Al,In]_*x*_Ga_1-*x*_)_2_O_3_, while at the same time being transparent to visible light and therefore insensitive to photon noise due to its wide band gap, demonstrating the application potential of this material system. Our simulations further show that the QWIPs efficiency critically depends on the QW thickness, making a precise control over the thickness during growth and a reliable thickness determination essential. We demonstrate that pulsed laser deposition yields the needed accuracy, by analyzing a series of (In_*x*_Ga_1-*x*_)_2_O_3_ QWs with (Al_*y*_Ga_1-*y*_)_2_O_3_ barriers with high-resolution X-ray diffraction, X-ray photoelectron spectroscopy (XPS) depth profiling, and transmission electron microscopy (TEM). While the superlattice fringes of high-resolution X-ray diffraction only yield an average combined thickness of the QWs and the barrier and X-ray spectroscopy depth profiling requires elaborated modeling of the XPS signal to accurately determine the thickness of such QWs, TEM is the method of choice when it comes to the determination of QW thicknesses.

## Introduction

Wide band gap semiconductors have gained increasing interest over the last decades due to their potential applications in high-power and high-temperature devices, such as ultraviolet light-emitting diodes, terahertz electronics, and others.^1^ Prominent members of this material class with established applications are SiC,^2^ ZnO,^3^ or GaN.^4^ With recent advancements in growth procedures, leading to improved stability and crystallinity,^5^ the orthorhombic κ-phase of Ga_2_O_3_ and its indium^6^ and aluminum^7,8^ alloys emerged as new potential candidates in this field. One remarkable property of these alloys is the tuneability of the band gap between 4.1 and 6.2 eV.^6,7^ Besides a direct usage of this varying band gap in, *e.g.*, deep ultraviolet photodetectors,^9^ the tuneable conduction band offsets in heterojunctions of these alloys could be employed in quantum well (QW) infrared photodetectors (QWIPs).^10^ The working principle is the following: discrete quantized energy levels, so-called sub-band edges, are formed within the QW, with their energy depending on the conduction band offset and the width of the well.^11,12^ Photons with an energy matching the energy difference between two sub-bands are absorbed within the QW and can be extracted as a current when a bias is applied.

The κ-phase of Ga_2_O_3_ is the most promising of the Ga_2_O_3_ polymorphs for such applications as it is the only one with a large spontaneous electrical polarization along its *c*-axis direction. The expected value of 24.2 μC/cm^2^ exceeds the one of established materials, such as GaN, by about 1 order of magnitude.^13^ This would allow for polarization doping, where the lowest QW sub-band can be populated without extrinsic doping by interface charges and corresponding band bending within the QW, arising from polarization discontinuities at the QW/barrier layer interface. This potentially increases the quality and performance of QWIP devices. Due to the wide band gap of the material, the QWIP devices can be designed completely transparent to and unaffected by light in the visible to the UV spectral range, allowing for, *e.g.*, direct integration in visors of firefighter helmets while omitting additional filter layers needed to reduce dark currents due to background light in conventional QWIPs. The possibility to fabricate high-quality QW superlattice heterostructures in the κ-modification has been shown recently.^14^ Simulations of the κ-([Al,In]_*x*_Ga_1-*x*_)_2_O_3_ systems yield potential detection wavelengths in the range of 1–100 μm, substantially increasing the range of materials typically applied in QWIPs nowadays, like AlGaAs/GaAs or AlGaN/GaN. However, the responsivity of a QWIP depends critically on the thickness of the QW (as sub-band energies change with QW thickness) and exhibits a maximum when the second sub-band is in resonance with the QW barrier because an excited electron has a higher probability of tunneling out of the QW and contribute to the photocurrent.^15^ Methods for precise thickness determination are therefore crucial.

By studying a series of κ-([Al,In]_*x*_Ga_1-*x*_)_2_O_3_ QWs with varying thicknesses, we demonstrate that depth-resolved X-ray photoelectron spectroscopy (XPS) can yield accurate thicknesses of QWs, but elaborated modeling of the XPS signal must be applied. This includes considering the inelastic mean free path of the photoelectrons as well as a Gaussian broadening due to the sputtering process. The thicknesses determined in this manner are verified by comparison to transmission electron microscopy (TEM) measurements and high-resolution X-ray diffraction measurements.

## Methods

### Sample Preparation

The investigated QW thin-film sample was deposited on a 10 × 10 cm^2^ c-sapphire substrate by pulsed laser deposition (PLD), employing a 248 nm KrF excimer laser (Coherent LPX Pro 305 F). The laser was focused to a size of about 2 × 6.5 mm^2^, which corresponds to a laser fluence of about 2.6 J/cm^2^ on the target surface. The sample consists of an about 120 nm thick κ-Ga_2_O_3_ buffer layer followed by an about 120 nm thick κ-(Al_*x*_Ga_1-*x*_)_2_O_3_ buffer layer deposited employing a target consisting of Ga_2_O_3_ and 35 at % Al_2_O_3_ in the mixture. Finally, a series of 5 κ-(In_*y*_Ga_1-*y*_)_2_O_3_/(Al_*x*_Ga_1-*x*_)_2_O_3_ QW structures were grown. A combinatorial PLD method was used to grow the QW layers using an elliptically segmented target consisting of Ga_2_O_3_ in the inner segment and Ga_2_O_3_ with 40 at % In_2_O_3_ in the outer segment.^16^ The radius of the circular laser spot track was fixed to 6 mm to obtain the targeted In-composition. For the barrier layer, the same target as for the κ-(Al_*x*_Ga_1-*x*_)_2_O_3_ buffer layer was used. The laser pulse number of the κ-(In_*y*_Ga_1-*y*_)_2_O_3_ QW layers was increased for each layer toward the surface of the sample from 50, 100, 150, 200 to 250 pulses. All targets additionally contained 1.5 or 2 wt % SnO_2_ in the mixture to facilitate the growth in the metastable κ-phase.^5^ The SnO_2_ only acts as a catalyst but is not incorporated into the thin-film layers in a significant amount. The layers were all grown at a growth temperature of about 610 °C, while an oxygen partial pressure of 0.002 mbar was chosen for the binary Ga_2_O_3_ buffer layer as well as the Al-containing layers and 0.006 mbar for the In-containing layers, respectively. A repetition frequency of 10 Hz was employed for the Ga_2_O_3_ buffer layer as well as the Al-containing layers, while for the In-containing QW layers, a lower frequency of 3 Hz was chosen. The targets were prepared, as described elsewhere.^14,16^ The growth of the sample in the κ-modification with a typical (001) orientation^16^ was confirmed by X-ray diffraction (Figure S1a, Supporting Information).

### X-ray Photoelectron Spectroscopy

The XPS measurements were performed at Humboldt-Universität zu Berlin, using a JEOL JPS-9030 setup with a base pressure of 3 × 10^–9^ mbar, employing the K_α_ radiation of a non-monochromated Mg X-ray source (*h*ν = 1253.6 eV) mounted with an angle of 54.7° with regard to the analyzer for excitation and a hemispherical analyzer with a pass energy of 100 eV for maximal sensitivity, to detect the kinetic energy of the emitted electrons under normal emission, using a 1 mm^2^ aperture. The samples were mounted with the conductive copper tape on two sides (outline visible in Figure S2 in the Supporting Information) to reduce charging. Still some static charging was observed due to the low conductivity of Ga_2_O_3_ and its alloys. We waited a sufficient time after each sputter cycle (∼5 min) under X-ray illumination to reach equilibrium and to avoid changes during the measurements. Depth profiles were obtained using a Kaufmann-type etching ion source, with an Ar pressure of 3 × 10^–4^ mbar using the minimal ion energy available of 300 eV to minimize sputter damage. No differential sputtering could be observed. The sputter crater had a diameter of about 1 cm, and the sputter rate was determined subsequently with a DEKTAK profilometer, as shown in Figure S2 in the Supporting Information Sensitivity factors were calculated by using the transmission function determined by measuring a sputter-cleaned Ag foil reference sample,^17^ the atomic cross sections after Scofield provided by JEOL (2.85 for O 1s, 0.574 for Al 2p, 20.47 for Ga 2p_3/2_, and 22.05 for In3d, all in barns with respect to C 1s), and the inelastic mean free path for inorganic compounds given by Seah and Dench.^18^ The integrated areas of the Ga 2p_3/2_, O 1s, In3d, and Al 2p core levels after Shirley background removal were used for quantification. The inelastic mean free path of the In3d electrons of 1.54 nm used for modeling in Figure 4 is calculated considering a stoichiometry of In_0.2_Ga_1.7_O_3.1_, as estimated from the thickest QW, using the average of the values obtained by the predictive formulas in the NIST electron IMFP database^19^ by Gries (G1 equation, 1.49 nm) and the TPP-2M equation (1.58 nm), assuming a density of 6.44 g•cm^–3^ (density of Ga_2_O_3_), a number of valence electrons per molecule of 24 (6•3 for oxygen and 3•2 for Ga/In), and a band gap of ∼4.6 eV [obtained from our previous work on (In_*x*_Ga_1-*x*_)_2_O_3_].^10^

### Transmission Electron Microscopy

The samples for TEM were prepared by focused ion beam (FIB) milling, depositing Pt for protection of the lamella and applying a final polishing step with an ion energy of 2 keV. The high-angle annular darkfield scanning transmission electron microscopy (HAADF-STEM) images were recorded with a JEOL JEM-2200FS using an acceleration voltage of 200 kV and an electron beam spot size of 0.7 nm.

### Quantum-Well Simulations

The band structure calculations and simulations of electron and hole wave functions were performed employing a code solving Poisson and Schrödinger equations self-consistently.^20^ The composition-dependent band gap of the alloy layers was taken as *E*_g_(*x*) = (4.92 ± 2.21*x*) eV, as determined in previous reports,^5−8^ where the + sign corresponds to the κ-(Al_*x*_Ga_1-*x*_)_2_O_3_ and the – sign to the κ-(In_*x*_Ga_1-*x*_)_2_O_3_ alloy system, respectively. As also determined in a previous report,^10^ the valence band maximum only varies insignificantly in energy with the alloy composition in both cases, such that the conduction band offset equals the band gap difference of the alloy layers. The spontaneous polarization of the alloy layers was linearly interpolated between the theoretical values for κ-Ga_2_O_3_ (*P*_sp_ = −24.2 μC/cm^2^), κ-Al_2_O_3_ (*P*_sp_ = −27.5 μC/cm^2^), and κ-In_2_O_3_ (*P*_sp_ = −52.5 μC/cm^2^), as given by Shimada.^13^ Piezoelectric polarization, induced by epitaxial strain due to lattice mismatch between the layers, was not considered for simplicity due to the negligible influence of this contribution compared to the spontaneous polarization differences. The static dielectric constant was taken as ε_*r*_ = 10.5 and the effective electron mass as *m*_eff_ = 0.34 *m*_e_ similar to monoclinic Ga_2_O_3_,^21−23^ the latter due to the similar curvature of the conduction band minimum for both polymorphs,^24,25^ and was considered independent of the alloy composition due to the unavailability of data for these quantities. Note that the values of the dielectric constant of κ-Ga_2_O_3_ vary largely in the literature and were reported from about 10 up to as large as 32,^26−28^ which directly affects the internal electric field and the slope of the associated additional triangular potential within the QWs. Similarly, also the effective electron mass of κ-Ga_2_O_3_ varies in the literature,^24,25,27,29,30^ which directly affects the energy levels within the QWs. The exact experimental determination of these quantities for the binary κ-Ga_2_O_3_ as well as the alloy systems is therefore crucial for the exact design of QWIP structures. The background doping was taken as *N*_D_ = 5 × 10^17^ cm^–3^ for the κ-Ga_2_O_3_ and κ-(In,Ga)_2_O_3_ QW layers, as previously estimated as net doping density for κ-Ga_2_O_3_-based Schottky barrier diodes,^31^ and *N*_D_ = 5 × 10^15^ cm^–3^ for the insulating κ-(Al,Ga)_2_O_3_ barrier layers. The simulated sample structures consist of a first 1000 nm thick κ-(Al,Ga)_2_O_3_ barrier layer with certain Al-composition on top of the substrate layer, then the κ-Ga_2_O_3_ or κ-(In,Ga)_2_O_3_ QW layer with variable thicknesses and a second 200 nm thick κ-(Al,Ga)_2_O_3_ barrier layer with the same composition as the first one on top of the QW. The barrier layers have been chosen sufficiently thick such that no surface or substrate interface effects have an influence on the simulations. The mesh for the calculations had a step size of 0.1 Å for all structures considered, and the donors were taken as fully ionized. The detection wavelengths were determined as follows: simulations were first performed for fixed In and Al alloy content for varying QW thicknesses in step sizes of 0.25 nm. The respective QW thickness for which the second QW sub-band is resonant or coincides with the energetically lowest conduction band minimum of the κ-(Al,Ga)_2_O_3_ barrier at the interface to the QW was then determined from the simulations, see, for example, Figure S3 for calculated band structures and associated wave functions where the QW is too thick, one where the correct QW thickness has been identified and one where the QW is too thin, respectively. It can be shown that such resonant configurations maximize the responsivity due to optimization of both transition probability as well as escape probability of excited carriers from the QW.^11,15,32^ The relevant detection wavelength was then calculated from the corresponding transition energy of the first to the second QW sub-level for this respective resonant thickness.

## Results

### Potential Use as Quantum-Well Infrared Photodetectors (QWIPs)

**Figure 1:**
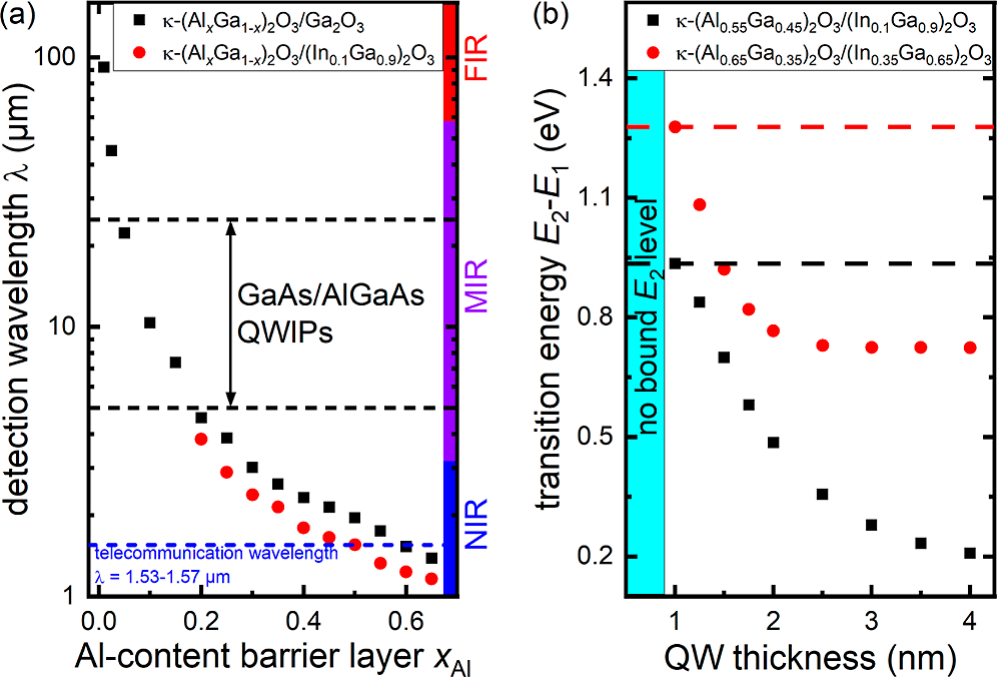
(a) Calculated detection wavelength of κ-(Al_*x*_Ga_1-*x*_)_2_O_3_/(In_*y*_Ga_1-*y*_)_2_O_3_ quantum-well infrared photodetectors (QWIPs) for different Al contents x_Al_ inside the barrier and two different In contents inside the quantum well (QW). (b) Transition energy E_2_-E_1_ as a function of QW thickness for two different Al and In contents. Dashed lines indicate the resonance energy.

As demonstrated previously, the energy offset between the conduction bands of κ-(Al_*x*_Ga_1-*x*_)_2_O_3_ and κ-(In_*y*_Ga_1-*y*_)_2_O_3_ can be tuned between 0 and 1.5 eV, depending on the Al and In content.^10^ If arranged in a QW structure, the inter-sub-band transitions lie in the infrared regime and can be used to detect incoming radiation, which is the fundamental principle of QWIPs.^11^ To yield a maximum response, the second energy state within the QW needs to coincide with the conduction band of the barrier, in order for excited electrons to be able to tunnel out of the QW and contribute more to the photocurrent. This is schematically illustrated in Figure S3. Typical wavelengths detectable with GaAs/Al_*x*_Ga_1-*x*_As QWs, one of the most commonly used material combinations for contemporary QWIPs, are in the range of 5–25 μm.^11,33^ The fundamental cutoff wavelength for n-type GaAs/Al_*x*_Ga_1-*x*_As is limited to about 3 μm for x∼1.^34^ Shorter wavelengths are possible but require the use of p-type GaAs/Al_*x*_Ga_1-*x*_As QWIPs, and also here a peak absorption at shortest wavelengths of only about 2 μm is given. This range can potentially be extended to both ends with the help of κ-(Al_*x*_Ga_1-*x*_)_2_O_3_/(In_*y*_Ga_1-*y*_)_2_O_3_, as shown in Figure 1a. Additionally, due to the large band gap of the material, the devices would be completely transparent in the visible spectral range. The possible detection wavelengths cover a large range in the far infrared, middle infrared, as well as near-infrared regime from about 100 μm down to about 1 μm, almost reaching the visible spectral range. With this, the range for thermal imaging as well as the important telecommunication wavelength at about 1.55 μm is included, providing the potential for precisely tuneable and transparent thermal detectors as well as transparent photodetectors for optical communication systems that are insensitive to photon noise in the visible regime and are able to operate at room temperature. As can also be seen in Figure S3, an additional triangular potential forms within the QW due to the internal electric field caused by the polarization discontinuities at the QW/barrier interfaces. The magnitude of this polarization discontinuity, and with this the internal electric field and depth of the triangular potential, depends on both the composition of the QW as well as the barrier layer and is especially sensitive to the In content due to the much larger spontaneous polarization of κ-In_2_O_3_ compared to both κ-Ga_2_O_3_ and κ-Al_2_O_3_.^13^ In the case of an In content of *x*_In_ = 0.1, the polarization difference and internal electric field increase with decreasing Al-content in the barrier layer in contrast to the case of a binary κ-Ga_2_O_3_ QW since both Al and In incorporation increase the magnitude of the spontaneous polarization of the alloy system. Below a certain critical Al-content in the barrier (*x*_Al_ = 0.15 for *x*_In_ = 0.1), the strength of the internal electric field is so large that the corresponding triangular potential dominates the wave functions and energy sub-levels in the QW, and no second bound level forms in the QW irrespective of QW thickness. This is the reason why no detection wavelengths are given for *x*_Al_ ≤ 0.15 in the case of the In-containing QW structure, as shown in Figure 1a. The critical Al-content increases with increasing In-content in the QW layer such that this effect needs to be considered in practical QWIP designs for the orthorhombic alloy systems. However, until now, no experimental values for the spontaneous polarizations of the orthorhombic alloy systems are available, such that their determination is of critical importance for the practical application of these alloy systems for QWIPs. Besides the formation of a second bound level, the spontaneous polarizations also determine the effectiveness of the polarization doping to populate the ground state of the QWs without extrinsic doping. As explained in the Methods section, also the experimental determination of the static dielectric constant as well as the effective electron masses of the orthorhombic alloy systems is crucial for exact QWIP simulations and design.

The transition energy of the QWs depends critically on their thicknesses, as experimentally demonstrated by Steel et al. for GaAs/AlGaAs^15^ and also predicted by our simulations for κ-(Al_*x*_Ga_1-*x*_)_2_O_3_/(In_*y*_Ga_1-*y*_)_2_O_3_, as shown in Figure 1b for two different aluminum and indium concentrations. The resonant transition energies are indicated by dashed lines. If the QW becomes too thin, no second energy level forms at all (see Figure S3c), whereas if the QW is too thick, the second energy level will be bound tightly within the QW and contribute significantly less to the photocurrent (see Figure S3a). With changes in transition energy of up to 0.5 eV/nm, it becomes clear that a good control of the growth process and proper means to accurately determine and control the thickness of the QWs are of paramount importance. In the next sections, we demonstrate by different techniques that PLD gives the precise control over the growth rate needed for such applications.

### X-ray Photoelectron Spectroscopy (XPS) Depth Profile

**Figure 2:**
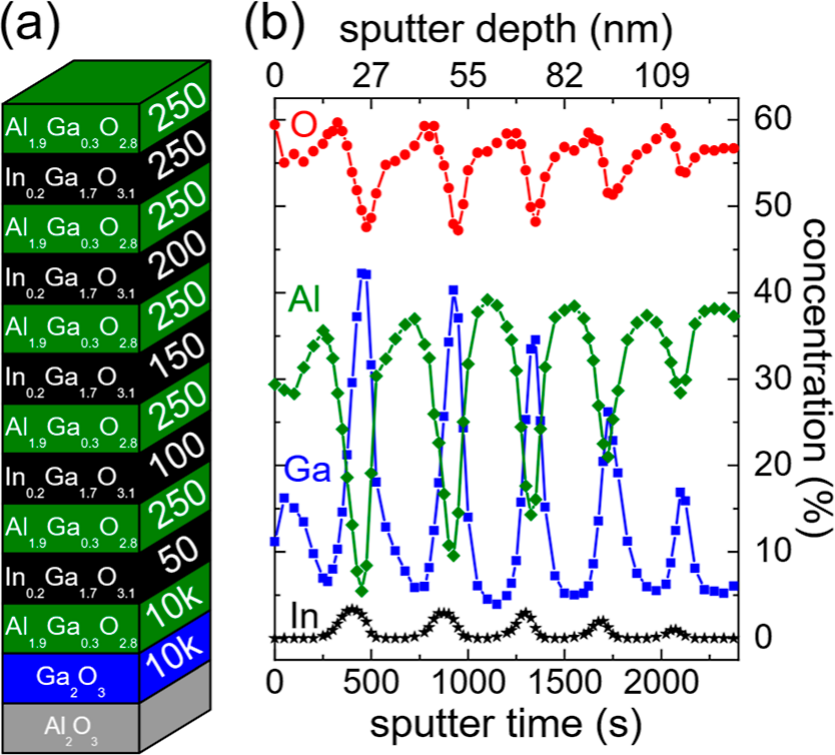
(a) Schematic sample structure. The numbers indicate the used PLD pulses for layer deposition. (b) XPS depth profile of the multi-QW κ-([Al,In]_*x*_Ga_1-*x*_)_2_O_3_ sample studied in this work. Sputter time was converted to sputter depth by calibrating the total sputter depth with a DEKTAK profilometer (see Figure S2).

The schematic sample structure and the XPS depth profile of a multi-QW structure, with varying pulse number used for the deposition of the various QWs, are shown in Figure 2. All XPS spectra are shown in Figure S4, and fits to selected spectra are shown in Figure S5 in the Supporting Information. A composition of the barrier layers of about Al_1.9_Ga_0.3_O_2.8_ was found. The composition of the QWs is hard to determine accurately due to an overlap of the signals from the barrier below but is estimated from the thickest QW to be In_0.2_Ga_1.7_O_3.1_ (the apparent decrease in oxygen inside the QWs seen in Figure 2b is due to the different inelastic mean free paths of gallium Ga2p_3/2_, aluminum Al2p, and oxygen O1s core levels). An intuitive fit of the QW In3d intensity *vs* sputter depth with Gaussian peaks (Figure S6) yields thicknesses of the QWs of (8.3 ± 0.2), (7.5 ± 0.2), (5.9 ± 0.2), (5.6 ± 0.3), and (5.1 ± 0.7) nm for 250, 200, 150, 100, and 50 PLD pulses, respectively.

### Transmission Electron Microscopy (TEM)

**Figure 3:**
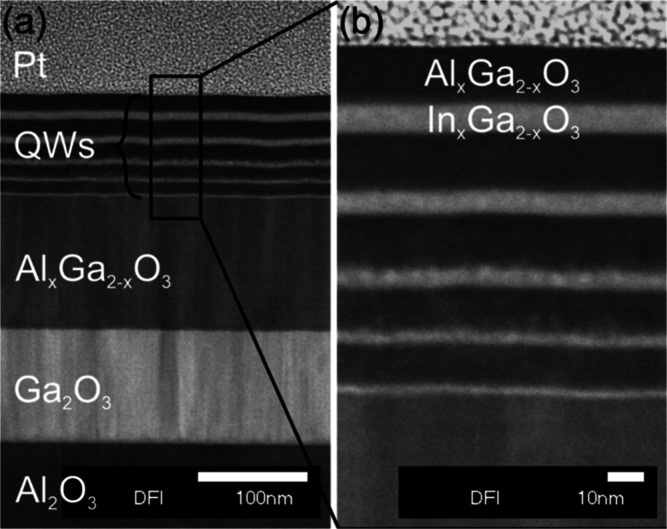
(a) Overview TEM image of the cross section of the whole sample stack. (b) Zoom into the QW region indicated by the black rectangle in (a), used to determine the QW thicknesses.

Figure 3a shows a TEM overview of the whole sample structure. On the bottom, one can see the sapphire (Al_2_O_3_) substrate, followed by a κ-Ga_2_O_3_ and a κ-(Al_*x*_Ga_1-*x*_)_2_O_3_ buffer layer, the multi-QW region, and Pt on top deposited during the TEM sample preparation by FIB. A zoom into the QW region is shown in Figure 3b. The thickness *d* of the QWs as a function of the number of PLD pulses *n* could be accurately determined from the high-resolution TEM measurement, resulting in a linear relation given by *d* = (0.033 ± 0.001 nm)•*n*. The abrupt interfaces between QWs and barriers are in agreement with the observation of superlattice fringes in XRD (see Figure S1b in Supporting Information), yielding an average double-layer thickness of one κ-(In,Ga)_2_O_3_/κ-(Al,Ga)_2_O_3_ layer stack in the QW superlattice structure of about 22 nm. Nevertheless, local variations in QW thickness as, *e.g.*, observed by STM by Chen et al. could still be present.^35^ The linear relation between pulse number and QW thickness is in apparent contrast to the thickness determined from a Gaussian fitting of the In3d intensity *vs* sputter depth (Figure S6), where the thickness saturates for small pulse numbers. The reason for this difference is discussed in the next section.

## Discussion

### Modeling of the QW X-ray Photoelectron Spectroscopy (XPS) Signal

**Figure 4:**
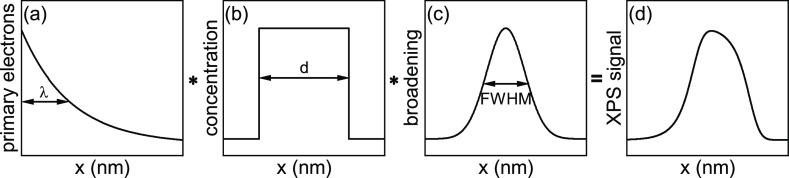
Different contributions of a QW to the measured XPS signal at different depths x. The depth distribution of primary electrons (exponential function described by the inelastic mean free path λ) (a) is convoluted with the actual elemental distribution of the QW of thickness d (b) and a broadening due to the sputtering process described by a Gaussian function with a full width at half maximum (FWHM) (c), which results in an asymmetric intensity distribution of the measured XPS intensity vs sputter depth (d).

**Figure 5:**
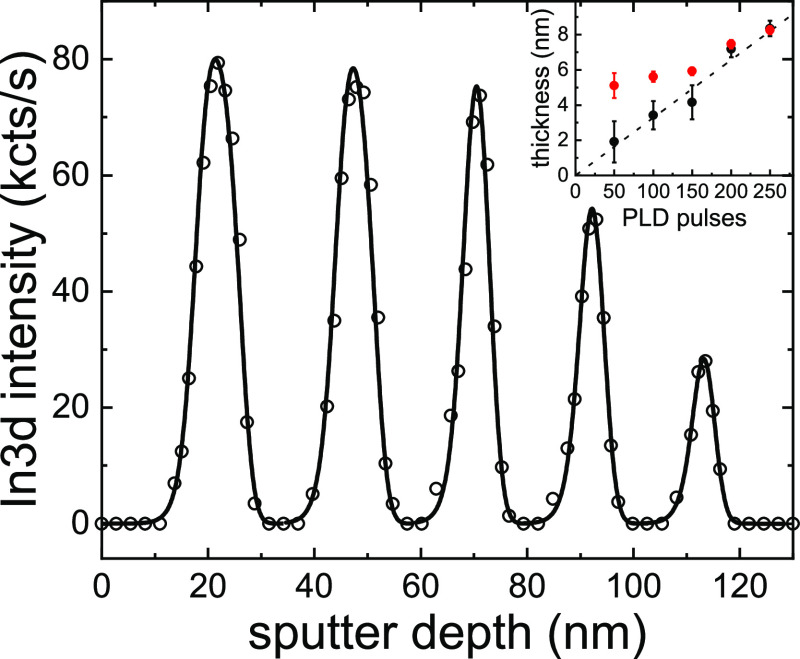
Measured In3d intensity (open circles) vs sputter depth and a fit (solid line) according to the procedure described in Figure 4 and in the main text. During the fitting procedure, a constant broadening due to the sputtering process was assumed. This results in a broadening of about 4 nm and QW thicknesses as shown in the inset (black dots), which are in good agreement with the thicknesses determined from TEM (dashed line). The thicknesses determined from Gaussian fits (Figure S4, Supporting Information) are shown as red circles for comparison.

As noted above, a simple Gaussian fitting of the In3d intensity *vs* sputter depth yields an overestimation of the determined thickness for very thin QWs. Similar effects were observed by Lisowski et al. for InGaN/GaN QWs.^36^ The reason for this is that the XPS signal is a convolution of multiple contributions, described in detail by Hofmann.^37^ Besides the actual elemental distribution, the signal is influenced by (1) the information depth characterized by the inelastic mean free path (IMFP, λ) of the photoelectrons in XPS and (2) a broadening due to the sputtering process, usually assumed as a Gaussian function characterized by a full width at half-maximum (FWHM). The IMFP λ is either experimentally determined from calculations based on optical measurements or by elastic-peak electron spectroscopy or, more often, theoretically predicted.^38^ Different models are available, which are discussed in detail by Powell *et al*.^39^ The IMFPs calculated with different models can vary by up to ∼20%.^40^ We estimated the IMFP of In3d electrons to 1.54 nm, using the NIST inelastic-mean-free-path database (details are given in the Methods section).^19^ The different contributions and the resulting signal are schematically shown in Figure 4 and given by the following equation^41^
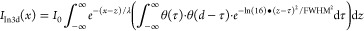
Here, θ(τ) is the Heaviside function. If we fit the In3d intensity *vs* sputter depth of the different QWs with this, as shown in Figure 5, leaving the thickness of the QWs and the constant FWHM of the broadening as fitting parameters, we obtain a broadening of about 4 nm and thicknesses of the QWs, as shown in the inset (black dots), which are in excellent agreement with the TEM results (dotted line). This is only possible because we analyze a series of QWs under the same sputtering conditions, demonstrating that certain prerequisites are needed for depth-resolved XPS to yield accurate thicknesses, highlighting that analysis must be carefully implemented for thickness determinations of QW structures. It should be mentioned that the use of a different IMFP for the modeling would also lead to different thicknesses obtained by the fitting, introducing another source of uncertainty. Other methods, like high-resolution X-ray diffraction on QW superlattice heterostructures (see Figure S1b in Supporting Information)^14^ or especially high-resolution TEM are therefore better suited for this purpose, if available and applicable.

## Conclusions

A series of PLD grown κ-([Al,In]_*x*_Ga_1-*x*_)_2_O_3_ QWs with varying thicknesses were investigated by high-resolution X-ray diffraction, XPS depth profiling, and TEM. We demonstrated that only an elaborated modeling of the XPS QW signal *vs* sputter depth, considering the inelastic mean free path of the emitted photoelectrons and a broadening due to the sputtering process, can yield correct thickness values. This holds especially for QWs only a few nanometer thick. TEM should be preferred for the purpose of QW thickness determination, if available. Simulations showed that the κ-([Al,In]_*x*_Ga_1-*x*_)_2_O_3_ QW structures have potential application as QWIPs. The detection wavelength was simulated and ranges between 1 and 100 μm, substantially extending the range of commercially available QWIPs nowadays. An increase of indium content can decrease the potential detection wavelength, while a decrease of aluminum content in the barrier layers can potentially increase the detection wavelength. Simulations further showed that the transition energies depend critically on the QW thickness. Our findings demonstrate that the sub-nanometer precision of PLD growth has the potential for the development of QWIPs based on κ-([Al,In]_*x*_Ga_1-*x*_)_2_O_3_ QWs.

## Supporting Information Available

XRD measurements, DEKTAK measurements for sputter rate determination, simulated QW energy levels, XPS spectra, and Gaussian fit of the indium intensity *vs* sputter depth (PDF)
