# Supplementary material for: Growth of κ-([Al,In]xGa1-x)2O3 Quantum Wells and Their Potential for Quantum-Well Infrared Photodetectors
Source: ACS Appl Mater Interfaces. 2023 Jun 6;15(24):29535–41. doi: 10.1021/acsami.3c02695 (PMC10288438; doi:10.1021/acsami.3c02695)
Supplement: Supplementary file 1 — am3c02695_si_001.pdf [file am3c02695_si_001.pdf]

Supporting information

Growth of κ-$([Al,In]_xGa_{1-x})_2O_3$ Quantum Wells and their Potential for Quantum Well Infrared Photodetectors

Thorsten Schultz[1,2,*], Max Kneiß[3], Philipp Storm[3], Daniel Splith[3], Holger von Wenckstern[3], Christoph T. Koch[2], Adnan Hammud[4], Marius Grundmann[3], Norbert Koch[1,2]

[1]*Helmholtz-Zentrum Berlin für Materialien und Energie GmbH, 14109 Berlin, Germany*

[2]*Humboldt-Universität zu Berlin, Institut für Physik & IRIS Adlershof, 12489 Berlin, Germany*

[3]*Universität Leipzig, Felix-Bloch-Institut für Festkörperphysik, 04103 Leipzig, Germany*

[4]*Fritz-Haber Institute of the Max-Planck Society, Department of Inorganic Chemistry, 14195 Berlin, Germany*

[*]E-mail corresponding author: thorsten.schultz@helmholtz-berlin.de

S1: XRD spectra

(a)

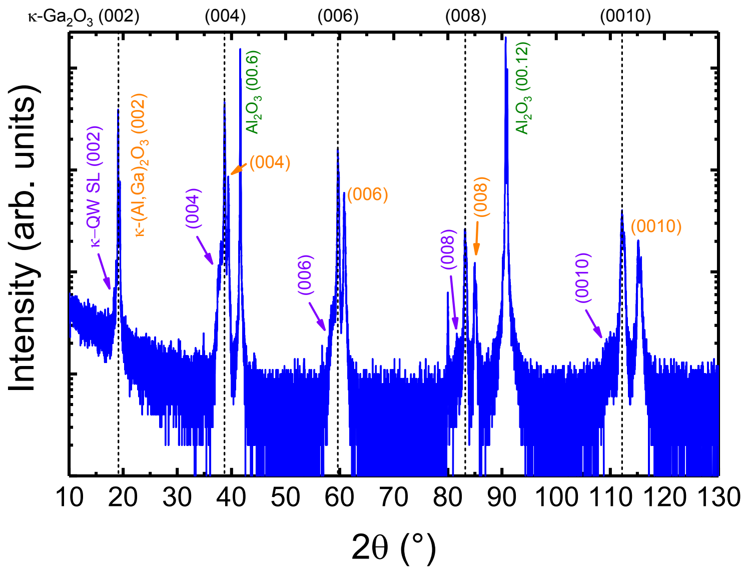

(b)

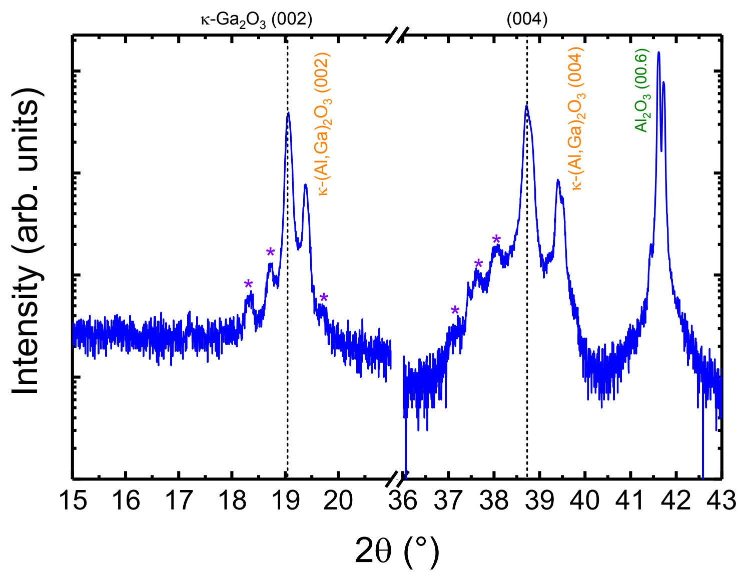

Figure S1: XRD 2θ-ω of a κ-$(In,Ga)_2O_3$/κ-$(Al,Ga)_2O_3$ quantum well superlattice (QW SL) layer with varying quantum well thickness increasing from bottom to top of the layer. The quantum well layer was deposited by pulsed laser deposition on a κ-$(Al,Ga)_2O_3$ buffer layer on top of a κ-Ga_2O_3 buffer layer on c-plane sapphire as described in the main text. (a) Complete 2θ range from 10-130°. Reflections from the substrate are indicated in green, while reflections from the κ-Ga_2O_3 buffer layer are indicated by dashed lines and indexed in black. Reflections indicated in orange stem from the κ-$(Al,Ga)_2O_3$ buffer layer. The reflections of the QW SL layer are located at the low angle side of the corresponding κ-Ga_2O_3 reflections and indicated in violet. Only κ-phase (00N) reflections could have been indexed corroborating a (001) oriented growth of all layers in the κ-modification. (b) Magnified view around the κ-Ga_2O_3 (002) and (004) reflections. Distinct superlattice fringes occurring due to the almost periodic structure of the QW SL layer are visible in this magnification and are indexed by violet asterisks. The separation of the fringes corresponds to an average double layer thickness of one κ-$(In,Ga)_2O_3$/κ-$(Al,Ga)_2O_3$ layer stack in the QW SL structure of about 22 nm and is in good agreement to the depth-resolved XPS data (~24 nm on average) and TEM data (~19 nm on average) presented in the main text. Note that the determination of the double layer thickness of a SL structure from the separation of SL fringes only strictly holds for periodic structures with identical thicknesses of each pair of layers in the SL structure and should only serve as crude estimation here. Nevertheless, the occurrence of SL fringes indicates abrupt and smooth interfaces as corroborated by the TEM measurements in the main text.

S2: Sputter rate determination

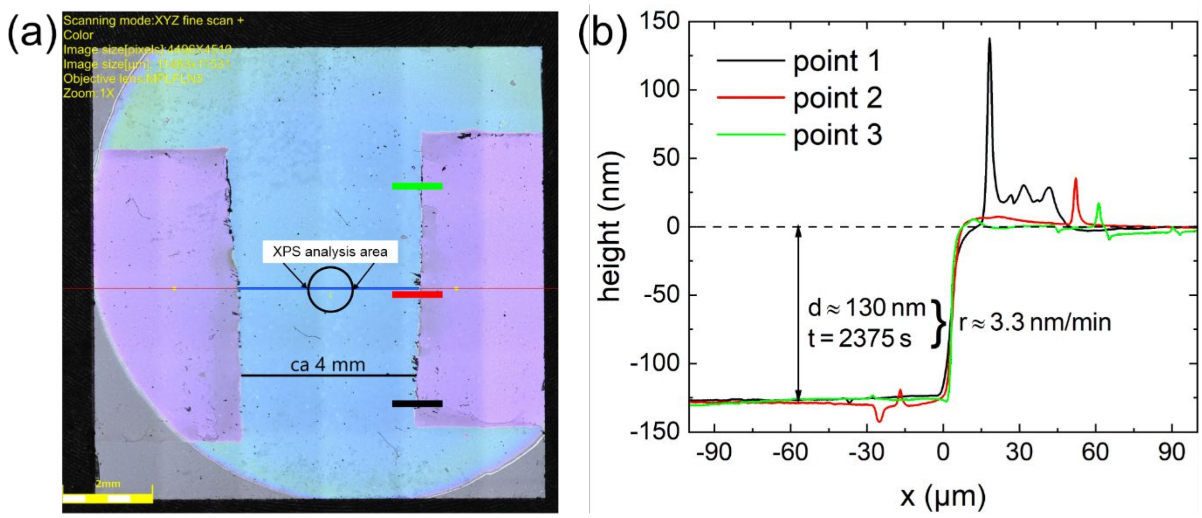

Figure S2: (a) Optical microscope image of the sample after depth profiling, indicating the X-ray photoelectron spectroscopy (XPS) analysis area with a diameter of ~1 mm. The area covered by the contacts during sputtering is purple to the left and right and the sputter crater is visible as a bright circle. (b) DEKTAK profiles measured at the positions indicated in (a). The measured steps yield a sputter rate *r* of about 3.3 nm/min.

S3: Simulated quantum well energy levels

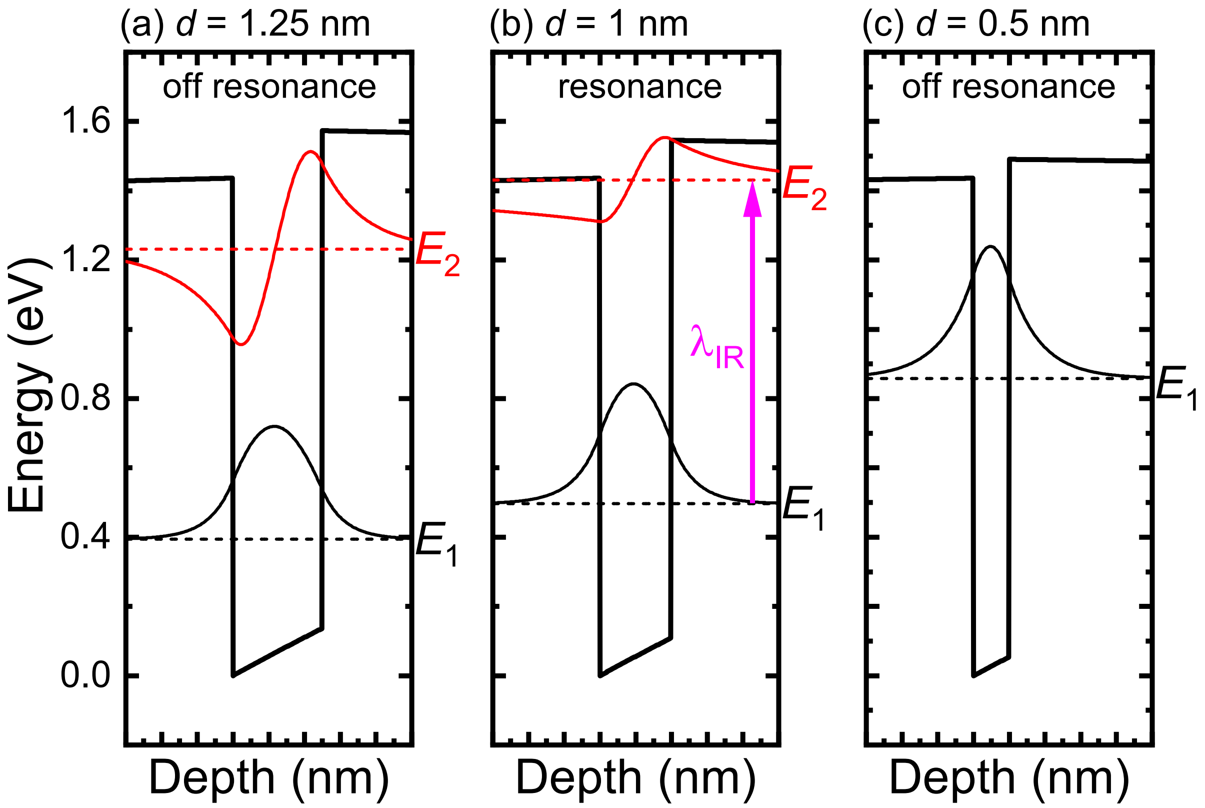

Figure S3: Simulated band diagram and energy states of κ-$(Al_{0.55}Ga_{0.45})_2O_3$/$(In_{0.1}Ga_{0.9})_2O_3$ quantum wells for three different thicknesses. (a) If the well is too thick, the second sub-band E_2 is bound within the quantum well and excited electrons can hardly escape the well and contribute to the photocurrent (off resonance). (b) For a suitable thickness, the second sub-band coincides with the quantum well barrier and excited electrons can more readily escape the well and contribute to the photocurrent (resonance). (c) If the well becomes too thin, the second sub-band moves out of the quantum well into the continuum (off resonance).

S4: XPS spectra

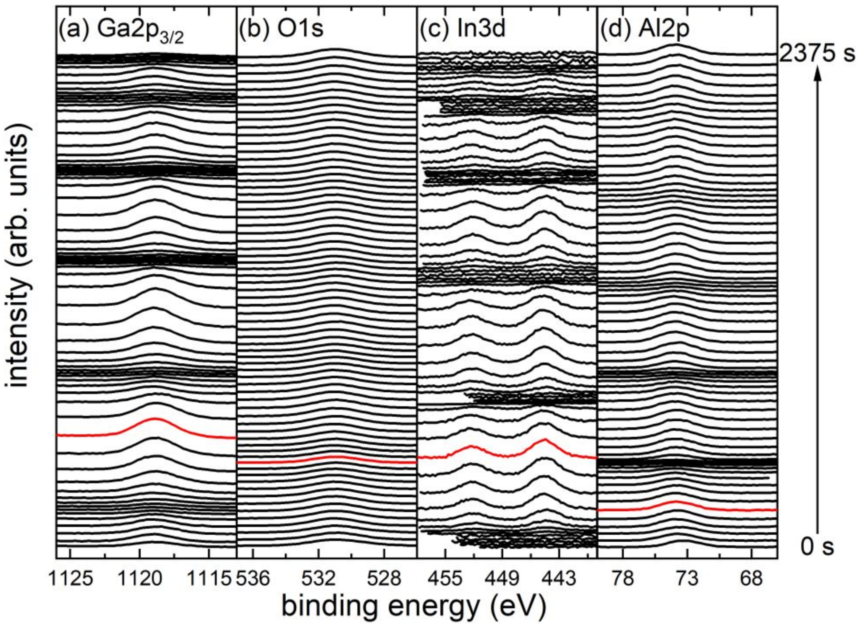

Figure S4: (a) $Ga2p_{3/2}$, (b) O1s, (c) In3d and (d) Al2p spectra of the XPS depth profile shown in Figure 1 in the main text. The sputter time increases from bottom to top. The red marked spectra are shown in Figure S5. Spectra were aligned to O1s at 531 eV binding energy for comparison.

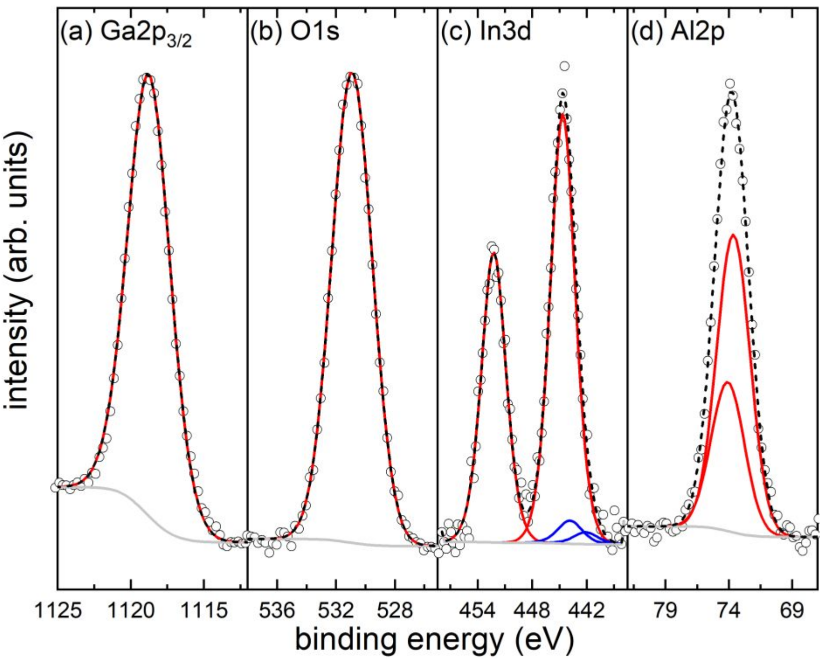

Figure S5: Selected (a) $Ga2p_{3/2}$, (b) O1s, (c) In3d and (d) Al2p core level as indicated in red in Figure S4. A Shirley background was applied and Voigt functions were used for fitting. The blue peaks in (c) are the Mg $K\alpha_{3,4}$ satellites of the $In3d_{3/2}$ peak due to the achromatic excitation source. The two red peaks in (c) and (d) are due to spin-orbit splitting.

S5: Gaussian fit of the indium intensity *vs* sputter depth

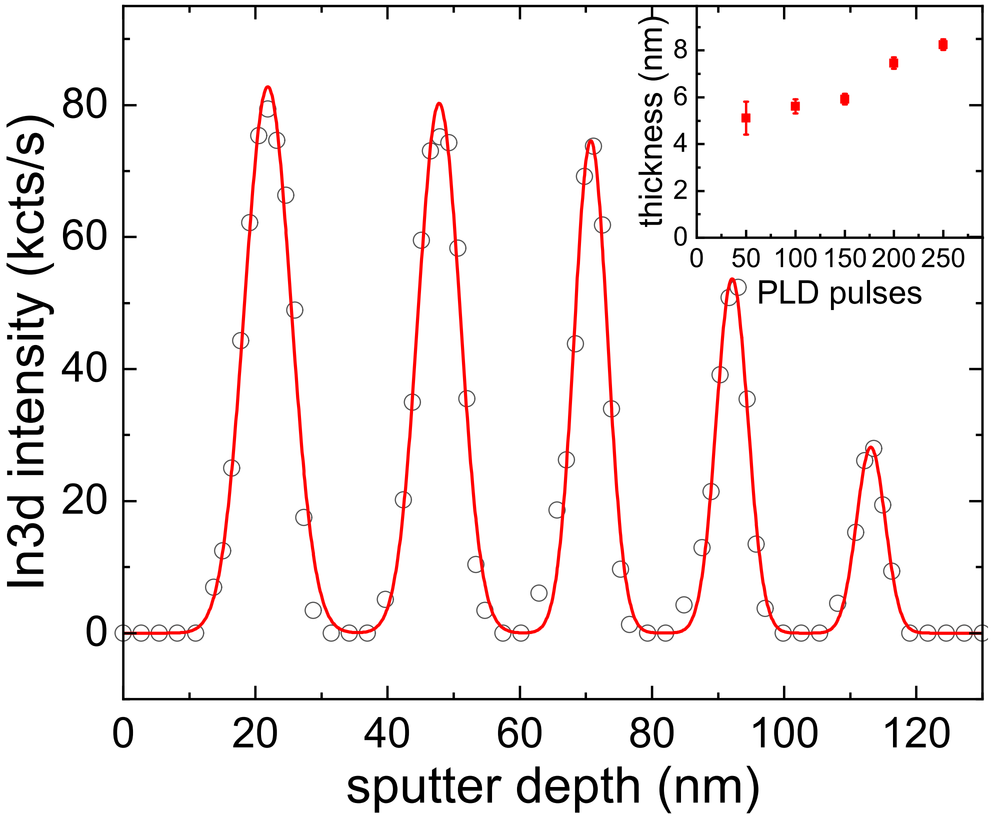

Figure S6: In3d signal intensity (dots) as a function of sputter depth, fitted with Gaussian peaks (red line). The thickness of the quantum wells determined from the Gaussian full width at half maximum (FWHM) is shown in the inset as a function of PLD pulses.
